# First Record of Comparative Plastid Genome Analysis and Phylogenetic Relationships among *Corylopsis* Siebold & Zucc. (Hamamelidaceae)

**DOI:** 10.3390/genes15030380

**Published:** 2024-03-20

**Authors:** Tae-Hee Kim, Young-Ho Ha, Hiroaki Setoguchi, Kyung Choi, Sang-Chul Kim, Hyuk-Jin Kim

**Affiliations:** 1 Division of Forest Biodiversity, Korea National Arboretum, Pocheon 11186, Republic of Korea; thkim3813@korea.kr (T.-H.K.);; 2 Graduate School of Human and Environmental Studies, Kyoto University, Kyoto 606-8501, Japan; 3 Division of Garden and Plant Resources, Korea National Arboretum, Pocheon 11186, Republic of Korea

**Keywords:** *Corylopsis*, plastid, phylogenetic analysis

## Abstract

*Corylopsis* Siebold & Zucc. (Hamamelidaceae) is widely used as a horticultural plant and comprises approximately 25 species in East Asia. Molecular research is essential to distinguish *Corylopsis* species, which are morphologically similar. Molecular research has been conducted using a small number of genes but not in *Corylopsis*. Plastid genomes of *Corylopsis* species (*Corylopsis gotoana, Corylopsis pauciflora*, and *Corylopsis sinensis*) were sequenced using next-generation sequencing techniques. Repeats and nucleotide diversity that could be used as DNA markers were also investigated. A phylogenetic investigation was carried out using 79 protein-coding genes to infer the evolutionary relationships within the genus *Corylopsis*. By including new plastomes, the overall plastid genome structure of *Corylopsis* was similar. Simple sequence repeats of 73–106 SSRs were identified in the protein-coding genes of the plastid genomes, and 33–40 long repeat sequences were identified in the plastomes. The Pi value of the *rpl33_rps18* region, an intergenic spacer, was the highest. Phylogenetic analysis demonstrated that *Corylopsis* is a monophyletic group and *Loropetalum* is closely related to *Corylopsis*. *C. pauciflora, C. gotoana*, and *C. spicata* formed a clade distributed in Japan, whereas *C. sinensis*, *C. glandulifera*, and *C. velutina* formed a clade that was distributed in China.

## 1. Introduction

The genus *Corylopsis* Siebold & Zucc. (Hamamelidaceae), commonly known as winter haze, comprises approximately 25 species of shrubs and small trees. This genus is restricted to the Northern Hemisphere, and many are found in East Asia, including Republic of Korea, Japan, China, and Taiwan [1]. *Corylopsis* has the following common morphological characteristics: deciduous shrubs with stellate pubescent branches; petiolate leaves, membranous or leathery blades, ovate to orbicular, margin serrate, raceme inflorescence, usually blooming before leaves; sepals 5, persistent or deciduous; petals 5 (rarely 4), yellow, ovate to spathulate [1,2]. *Corylopsis* species are commonly used as ornamental plants because of their attractive, yellow-flowered racemes in early spring [3]. In addition, it is used as a medicinal material; for example, *C. coreana* decreases the factor-induced generation of reactive oxygen species, and *C. sinensis* contains bergenin, which is a traditional Chinese medicinal material [3,4,5,6].

The classification of *Corylopsis* is controversial among botanists. In 1930, Harms suggested the intrageneric classification of the genus *Corylopsis* for the first time [1]. He divided the genus into five sections (Henryanae, Multiflorae, Pauciflorae, Spicatae, and Manipurenses) based on morphological characteristics, such as whether the ovary and hypanthium are fused, petal number, and nectary shape and number [1]. However, a heterogeneous section existed because the relationships between *Corylopsis* species that were more phenotypically similar could not be identified. Morley and Chao [1] explained that *Corylopsis* was divided into two groups, the Himalayan and Chinese, which were subdivided into continental and offshore groups. Furthermore, they provided a description, a new classification, and, by extension, a key to *Corylopsis*. In 2008, Yamanaka et al. [7] described the morphological characteristics of four species of *Corylopsis* distributed in Japan. The suggested morphological traits included leaf blades, inflorescences, stamens, stigmas, and staminodes. The identification of target species formed through morphological traits is often difficult, and recent phylogenetic analyses using molecular data, especially plastid genomes, are in progress [8,9,10,11,12].

Plastids, which are semi-autonomous organelles in plants, participate in the processes of photosynthesis and biosynthesis and range in size from 120 to 160 kb in general land plants [13,14,15]. The typical structure of the plastid genome consists of four parts in which two inverted repeats (IRs) divide the boundary between the large single-copy (LSC) and small single-copy (SSC) regions. Due to the high conservation of plastid protein-coding gene composition, reconstructing phylogenetic relationships among taxa is essential [16,17,18]. Furthermore, it is useful for inferring biogeography, molecular evolution, and age estimation [19,20]. With the development of next-generation sequencing (NGS), genomic data can be obtained quickly and easily. Consequently, more genes can be used for phylogenetic analysis, and the relationships between taxa can be reconstructed through high-resolution analysis.

As the morphological characteristics of *Corylopsis* species are similar and difficult to distinguish, molecular phylogenetic studies on this genus are necessary. Although Wang et al. [3] reconstructed the phylogenetic relationships of Hamamelidaceae using the plastid genome, the detailed phylogenetic relationships within the genus *Corylopsis* are unknown. Currently, the plastomes of seven species in the genus *Corylopsis* are registered in GenBank [3,21,22,23,24,25]. In this study, we aimed to (1) construct an unknown plastid genome of three species of *Corylopsis* to identify the phylogenetic relationships within Hamamelidaceae, (2) investigate repeats to propose DNA markers, and (3) perform a comparative examination of the plastid genome of Corylopsis and assess the phylogenetic associations.

## 2. Materials and Methods

### 2.1. Plant Material and DNA Extraction

Fresh leaves of *C. gotoana*, *C. pauciflora*, and *C. sinensis* were collected from fields in Japan, the Republic of Korea, and China (Table S1). All voucher specimens were deposited in the Herbarium of Korea National Arboretum with the collection numbers coryJ4 (*C. gotoana*), ESK22-086 (*C. pauciflora*), and coryc-2 (*C. sinensis*). After the leaves were dried with silica gel, the total genomic DNAs were extracted using a DNeasy Plant Mini Kit (Qiagen Inc., Valencia, CA, USA).

### 2.2. Genome Assembly and Annotation

A DNA library with an insert size of 550 bp was prepared, and next-generation sequencing (NGS) was performed using the Illumina MiSeq sequencing system at Macrogen Inc. (Seoul, Republic of Korea). Total raw reads were imported and trimmed with a 2% error probability limitation using Geneious Prime ver. 2019.0.4 to remove poor-quality reads [26]. The processed reads were assembled through ‘map to reference’ with *C. coreana* (GenBank accession no. NC_040141), which was used as a reference. Reads that were assembled into the reference genome were subjected to De novo assembly to create a scaffold contig. De novo assembly was conducted to reassemble the contigs using Geneious Prime [26]. The gene content and order were annotated using the Geseq tool and Geneious Prime [26,27].

### 2.3. Comparative Plastid Genome Analysis of Corylopsis

Ten plastid genomes of *Corylopsis*, three plastid genomes produced in this study, and seven plastid genomes provided by GenBank were compared. Using Geneious Prime ver. 2019.0.4, the GC content was calculated and compared. The mVISTA program in LAGAN mode was employed to analyze the entire plastome sequences of *Corylopsis*, with the annotation of *C. coreana* (GenBank accession no. NC_040141.1) as the reference [28,29]. IRscope was used to compare and illustrate the boundaries of inverted repeat (IR) and single copy (SC) sequences for *Corylopsis* species [30]. Relative synonymous codon usage (RSCU) for the CDS of the 10 *Corylopsis* cp genomes was calculated using DAMBE v 7.3.32 [31]. When RSCU > 1, this codon is used at higher frequencies than expected, and RSCU < 1 indicates the opposite. In addition, only genes in the IRA that were repeated in the IR regions were used.

### 2.4. Repeats and Nucleotide Diversity Analysis

Simple sequence repeats (SSRs) within *Corylopsis* were identified using MicroSAtellite ver. 2.1 (MISA). [32,33]. For MISA, we set the following parameters: ten for mononucleotides, five for dinucleotides, four for trinucleotides, and three for tetra-, penta-, and hexanucleotide SSR motifs. Long repeat analysis was conducted using the REPuter software with the following parameters: a minimal repeat size of 30 bp and a Hamming distance of 3 [34]. The nucleotide diversity (Pi) of cp genomes was examined using the DnaSP v. 6.0 program, which analyzed aligned sequences from 10 *Corylopsis* plastid genomes [35,36]. Pi values were calculated using a window length of 100 bp and a step size of 25 bp.

### 2.5. Phylogenetic Analyses

To investigate the phylogenetic relationships within *Corylopsis,* including the three newly sequenced plastid genomes, 20 cp genome sequences were obtained from NCBI. *Liquidambar styraciflua* (GenBank accession No. NC_046938) and *Liquidambar orientalis* (GenBank accession no. NC_046937) were designated as the outgroups. For phylogenetic analysis, 79 protein-coding genes were extracted and aligned using MAFFT ver. 7.313 with the default alignment parameters of the Phylosuite ver. 1.2.2 program [35,37]. The gaps present in the data were considered as missing values. Maximum parsimony (MP), maximum likelihood (ML), and Bayesian inference (BI) methods were used to analyze phylogenetic relationships. The MP analysis was performed using PAUP* v4.0a with equally weighted and unordered characters [38]. A heuristic search was employed to select the most parsimonious trees, which involved branch-swapping, tree bisection-reconnection (TBR), and MulTrees, allowing ten trees to be retained at each step. Bootstrap analyses comprising 1000 pseudoreplicates were performed to determine individual support values for each clade.

ModelFinder was used to determine the best model for ML and BI analyses. The best model for the concatenated data was GTR+F+I+G4, chosen according to Akaike’s information criterion (AIC) [39]. ML analysis was performed with 5000 replicates of ultrafast bootstrapping using the IQ-TREE web server [40]. MrBayes v3.2.6 was used for BI analysis [41]. Markov chain Monte Carlo (MCMC) algorithms were run for two million generations and sampled every 100 generations. In total, 25% of the generations were discarded as burn-ins. In Phylosuite, ML and BI analyses were performed using programs like ModelFinder, IQ-TREE, and MrBayes [37]. The phylogenetic trees were visualized using FigTree v1.4.4 (http://tree.bio.ed.ac.uk/software/figtree/ (accessed on 7 Decemeber 2023)).

## 3. Results

### 3.1. Plastid Genome Structure and Comparative Analysis of Corylopsis

**Figure 1:**
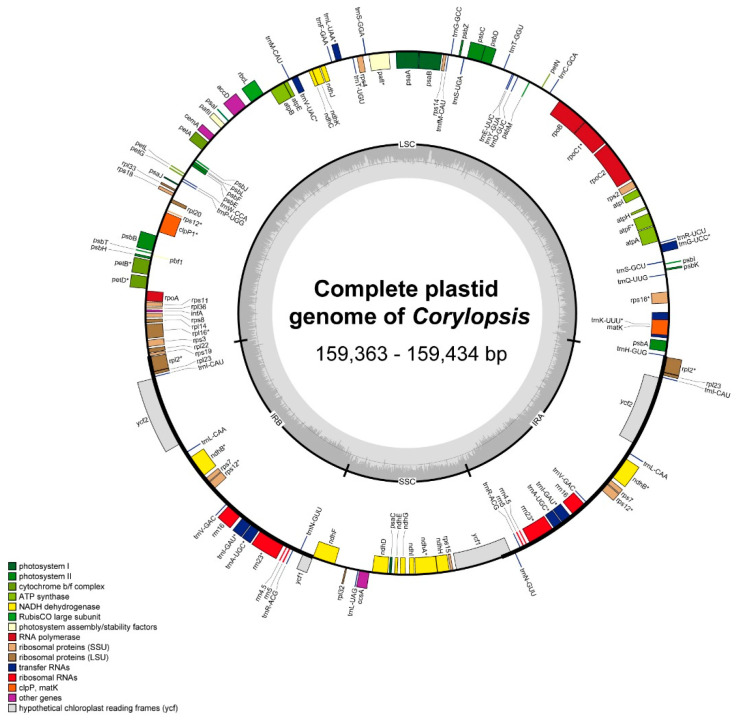
The complete plastid genome of *Corylopsis* manufactured in this study. Genes located within the inner portion of the circular structure are transcribed in a clockwise direction, whereas those positioned on the outer side are transcribed counterclockwise. The dark gray shading within the inner circle indicates the GC content, while the light gray represents the AT content. Various colors are used to indicate distinct functional genes. Genes containing intron are denoted with an asterisk (*).

Three plastid genomes of *Corylopsis* were obtained using NGS. The sequenced genomes had a quadripartite structure common to angiosperms, ranging from 159,363 bp (*C. pauciflora*) to 159,434 bp (*C. gotoana*) (Figure 1 and Figure S1). Ten plastid genomes of *Corylopsis* species produced in this study and from GenBank were obtained and analyzed. Among the *Corylopsis* species, the length of *C. multiflora* var. *nivea* was the smallest (158,993 bp), and *C. spicata* was the largest (159,507 bp) (Table 1). The overall GC contents of all *Corylopsis* were distinct at 38.0% (LSC, SSC, and IR were 36.1%, 32.6–32.7%, and 43.1%, respectively). The plastid genomes of the *Corylopsis* species contained 79 protein-coding genes, 30 tRNAs, and 4 rRNA genes (Table S2). Among these genes, 15 (*atpF*, *ndhA*, *ndhB*, *petB*, *petD*, *rpl2*, *rpl16*, *rpoC1*, *rps16*, *trnA-UGC*, *trnG-UCC*, *trnI-GAU*, *trnK-UUU*, *trnL-UAA*, and *trnV-UAC*) contained only one intron, and 3 (*clpP1*, *pafI*, and *rps12*) contained two introns. In addition, 17 genes (*ndhB*, *rpl2*, *rpl23*, *rps7*, *rps12*, *ycf2*, *trnA-UGC*, *trnI-CAU*, *trnI-GAU*, *trnL-CAA*, *trnN-GUU*, *trnR-ACG*, *trnV-GAC*, *rrn16*, *rrn23*, *rrn4.5*, and *rrn5*) were replicated in the IR regions. *rps12* was recognized as a trans-spliced gene, with its 5′ end located in the LSC region and its 3′ end in the IR regions.

**Figure 2:**
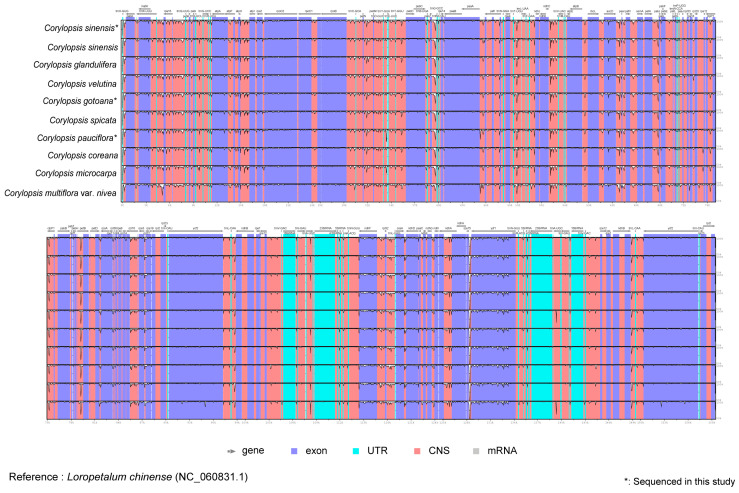
Using mVISTA plots, the alignment of plastid genome sequences was conducted to evaluate the percent sequence identity of the plastid genomes of 10 *Corylopsis* species, with *Loropetalum chinense* (NC_060831.1) serving as the reference. The *x*-axis represents the coordinate in the plastid genome, while the *y*-axis represents the average percentage of sequence similarity in the aligned regions, which ranges from 50% to 100%. Genome regions are categorized as protein-coding, rRNA-coding, tRNA-coding, or conserved noncoding sequences (CNS).

**Figure 3:**
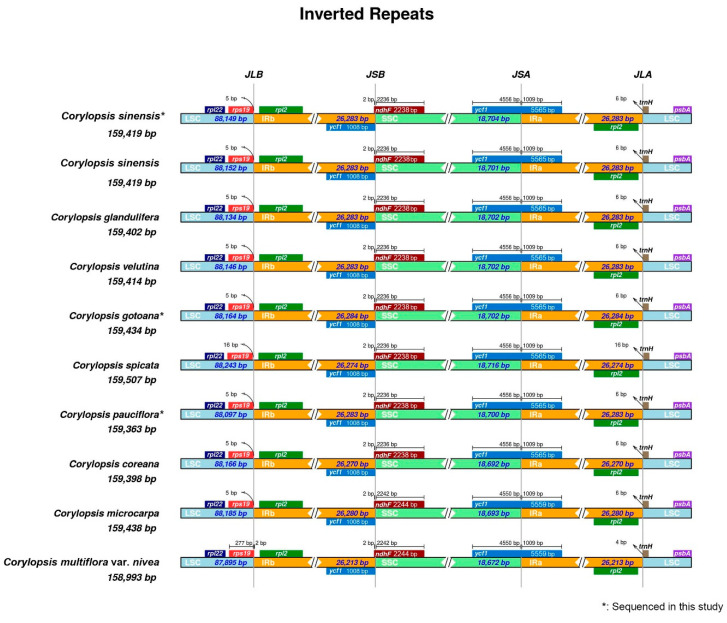
Comparison of LSC, SSC, and IR region boundaries in the plastomes of *Corylopsis*. JLB (IRB/LSC), JSB (IRB/SSC), JSA (IRA/LSC), and JLA (IRA/LSC) represent the junction sites between two adjacent regions in the genome.

In the mVISTA analysis, the complete plastid genomes of the 10 species were compared to the plastids of *Loropetalum chinense* as a reference (Figure 2). Overall, the plastid genomes were similar and conserved. In addition, the boundaries of the IRs were investigated (Figure 3). Most genes were preserved; however, *rps19* in *C. multiflora* var. *nivea* was located only at the junction between the LSC and IRb regions.

**Figure 4:**
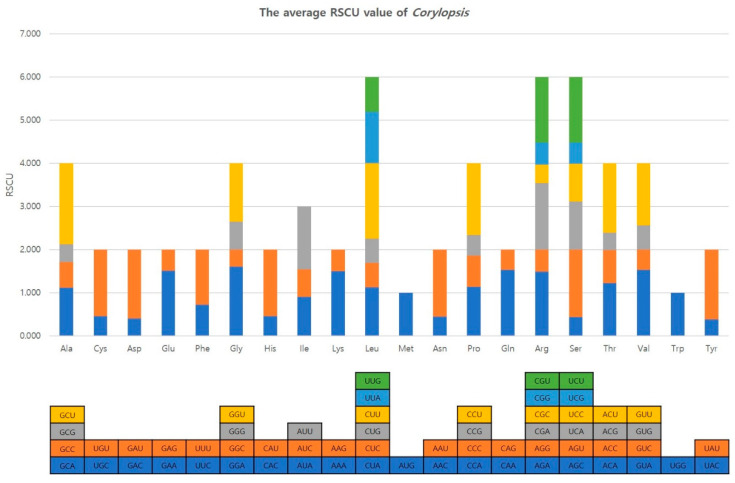
The average RSCU value of *Corylopsis*.

The relative synonymous codon usage (RSCU) of *Corylopsis* plastomes was computed using all protein-coding genes (Figure 4 and Table S3). The analysis confirmed that *Corylopsis* plastid genomes contained 61 codons encoding 20 amino acids. A total of 22,146–22,815 codons exist. Of the 61 codons, 29 had RSCU values greater than one. Methionine and tryptophan had a single codon. All codons had an RSCU > 1 end in the A/U at the third nucleotide position (except for UCC, which encodes Ser in *C. coreana*). On the other hand, out of the codons with RSCU values of one or less, only one had an A/U ending, whereas 31 codons had a G/C ending. In *C. coreana*, two Ser-encoding codons had a bias different from that in other species; when encoding Ser, UCC was used more often than UCA. The exact values for each species of *Corylopsis* are shown in Table S3.

### 3.2. Repeat Analysis and Nucleotide Diversity Assessment

**Figure 5:**
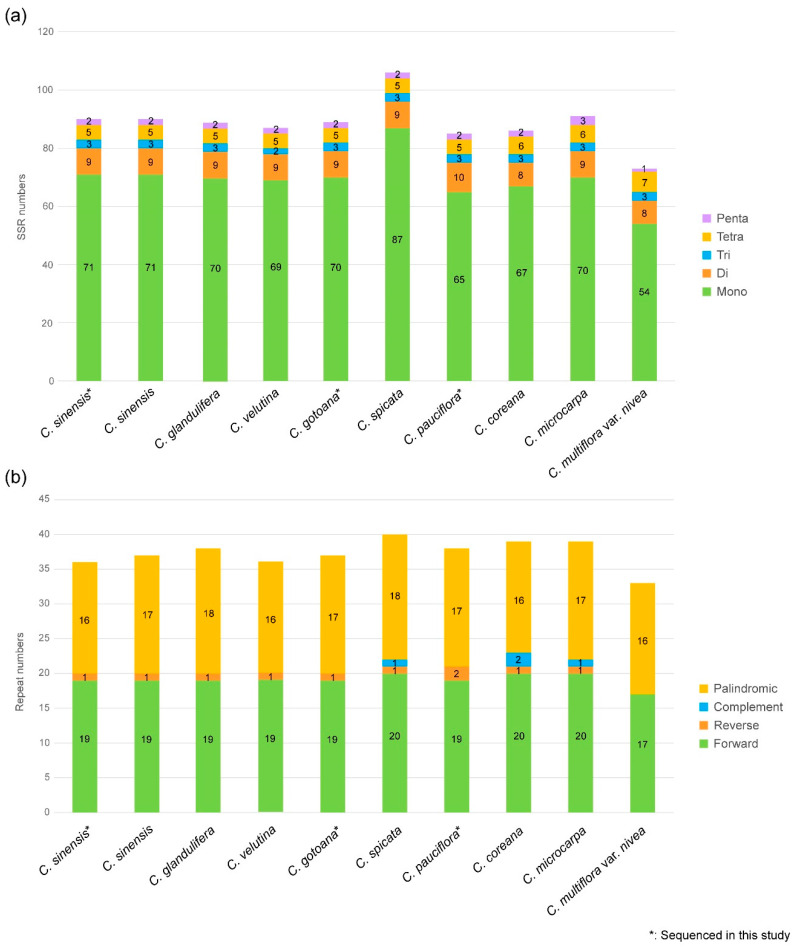
Analyses of repeats in the 10 plastid genome of *Corylopsis*. (**a**) The number of SSR motifs in *Corylopsis*. (**b**) Number of different repeat types in *Corylopsis*.

Simple sequence repeats (SSRs), also known as microsatellites, are short repeats composed of 1–6 nucleotide sequences of DNA segments within the genome. The SSRs for *Corylopsis* are shown in Figure 5a. As a result of the analysis, *Corylopsis* had 88 SSRs, and the species with the most number of SSRs was *C. spicata*, and *C. multiflora* var. *nivea* exhibited the lowest number of SSRs. This is because the number of mononucleotides is relatively large and small compared to that in other species. Among SSRs of *Corylopsis*, mononucleotides occupied the largest proportion (78.33%), most of which were A/T mononucleotides, followed by dinucleotide SSRs (10.05%), tetranucleotide SSRs (6.09%), trinucleotide SSRs (3.27%), and pentanucleotide SSRs (2.26%) (Figure 5a and Table S4).

In addition, we investigated forward, reverse, complement, and palindromic repeat sequences within the plastid genome of *Corylopsis* (Figure 5b and Table S5). Although the IR regions were repeated sections, the analysis results excluded them from Figure 5b. The average number of repeats in *Corylopsis* was 37, with a minimum of 33 repeats (*C. multiflora* var. *nivea*) and a maximum of 40 (*C. spicata*). Most of these were composed of forward and palindromic repeats. Complement repeats were found in only three species, *C. spicata*, *C. coreana*, and *C. microcarpa*, of which *C. coreana* had two complement repeats. Most species had one reverse repeat, whereas *C. pauciflora* had two reverse repeats. However, *C. multiflora* var. *nivea* confirmed the absence of reverse repeats.

**Figure 6:**
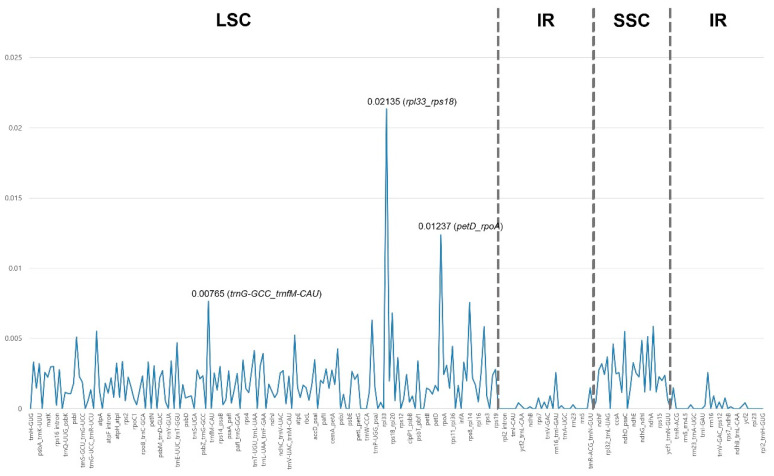
Nucleotide diversity (Pi) values in 10 *Corylopsis* species plastid genomes. The dashed lines demarcate the boundaries of the LSC, IR, and SSC regions.

To identify phylogenetically divergent hotspots, a nucleotide diversity (Pi) analysis of the complete plastid genome of *Corylopsis* was performed (Figure 6 and Table S6). Variable sites in the IR region were more conserved than those in the SC region. The Pi value was the highest at 0.02135 (*rpl33*_*rps18*), followed by 0.01237 (*petD*_*rpoA*), 0.00765 (*trnG-GCC*_*trnfM-CAU*), and 0.00756 (*rps8*_*rpl14*), all of which were intergenic spacers. Within the protein-coding genes, *ndhE* exhibited the highest Pi value (0.00327), followed by *rps16* (0.003), *rps19* (0.00279), and *ndhF* (0.00275).

### 3.3. Phylogenetic Analyses of Corylopsis and Related Taxa

Phylogenetic analyses were conducted using concatenated 79 protein-coding genes, employing maximum parsimony (MP), maximum likelihood (ML), and Bayesian inference (BI) methods. Consistently, all three tree constructions displayed identical topologies, with high support values at each node.

**Figure 7:**
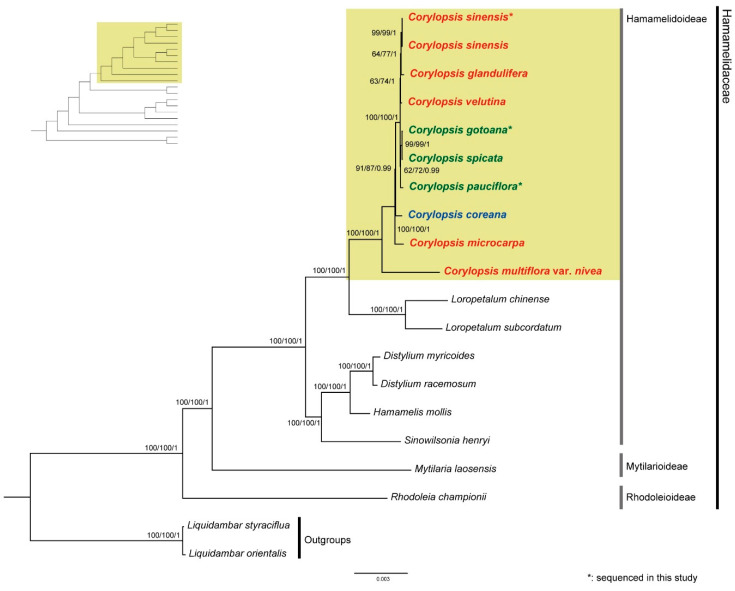
The ML tree of 20 species based on protein-coding genes. Above the nodes are MP bootstrap values, ML bootstrap values, and BI posterior probabilities (PP). The red, blue, and green colors indicate species distributed in China, the Republic of Korea, and Japan, respectively.

Twenty species were used to reconstruct phylogenetic relationships within Hamamelidaceae (Figure 7). *Liquidambar styraciflua* and *L. orientalis* (*Altingiaceae*) were designated as outgroups. In Hamamelidaceae, Rhodoleioideae, including *Rhodoleia*, was sister to the remaining taxa. Hamamelidoideae, including *Sinowilsonia*, *Hamamelis*, *Distylium*, *Loropetalum*, and *Corylopsis*, were located in the upper clade and divided into two major clades: *Sinowilsonia*, *Hamamelis*, and *Distylium*, *Loropetalum*, and *Corylopsis*. The genus closest to *Corylopsis* was identified as *Loropetalum,* with a high support value.

The genus *Corylopsis* was monophyletic, and most nodes showed high levels of support. *C. multiflora* var. *nivea* was sister to the remaining *Corylopsis*, followed by *C. microcarpa* and *C. coreana*. *C. pauciflora*, *C. spicata*, and *C. gotoana* formed one clade, and *C. pauciflora* was confirmed to be the sister of the other species, with relatively weak support values in the MP and ML analyses (62 and 72, respectively). Another clade consisted of *C. velutina*, *C. glandulifera*, and *C. sinensis*, where *C. velutina* was sister to the other species.

## 4. Discussion

### 4.1. Comparison of Plastid Genomes and Characteristics of Corylopsis

Several studies have used plastid genomes to identify the characteristics of target species and comprehend phylogenetic relationships within taxa [16,17,18]. This study confirmed that the plastid genome of *Corylopsis* has a typical quadripartite structure. The plastomes of the three species of *Corylopsis* completed in this study were based on Illumina MiSeq sequencing and had a length of 159,363 bp (*C. pauciflora*) to 159,434 bp (*C. gotoana*) and an average GC content of 38.0%. The GC content of the IR regions is relatively higher than that of the SC regions, implying that fewer AT contents have relatively weak hydrogen bonds. Consequently, evidence exists that IR regions are better preserved than SC regions (Figure 2 and Figure 6, Table 1) [20,42,43,44,45]. The IR region is commonly found duplicated in most plastid genomes. Due to this arrangement, it is thought that the IR region provides structural stability to the circularized plastomes [46]. In addition, these repetitions can aid in limiting gene movement and rearrangement, thus contributing stability. In the case of transgenes, insertion into the IR regions is necessary to double the copy number and enhance homoplasmy to strengthen the selection pressure [47]. This is because when transgenes are inserted into the IR region, they are also inserted into the other copy. Indeed, homoplasmy, which refers to the integration of foreign genes into all plastid genomes, and increased levels of polymer transcripts were detected only within the IR region, with no such observations in LSC transgenic plants [48]. Because of the comparison of the plastome structure and mVISTA analysis, it was confirmed that the characteristics such as GC content, genome length, and content of genes were generally similar and well conserved within *Corylopsis* (Figure 2, Table 1 and Table S2). Through IR scope analysis, the IR boundaries of the three plastid genomes created in this study were well conserved, similar to those of other species. Among the other species, a notable difference was observed in *C. multiflora* var. *nivea*, where the *rps19* gene was present at the boundary between the LSC and Irb. The fate of genes located at the boundaries of each region also depends on the extension or contraction of specific regions of the plastome, which can be used for the phylogenetic classification of taxa [49,50,51,52]. For example, in the genus *Camellia* (Theaceae), variations in the length of the IR regions due to various indels in the plastome have been observed [51]. The extension or contraction of at least one to seven IR regions has been observed within Apioideae (Apiaceae), which explains the pattern of genetic evolution in Apioideae [52]. *C. multiflora* var. *nivea* may be considered an evolutionary phenomenon in *Corylopsis*, although the plastid genome structure did not show evident differences, as in the aforementioned taxa. The location of the *rps19* gene of *C. multiflora* var. *nivea* revealed in this study can provide the basis for understanding the evolutionary patterns of *Corylopsis* in the future.

Synonymous codons encode the same amino acids in many eukaryotes but occur at different frequencies, which is referred to as codon bias [45,53,54]. Codon bias is determined by various factors such as base composition, gene length, and amino acid hydrophobicity, and is involved in regulating gene expressions and increasing translation accuracy and efficiency [54,55,56,57,58]. This study confirmed that the RSCU values of 29 out of 61 codons encoding amino acids in *Corylopsis* were more than one, indicating codon bias (Figure 4 and Table S3). Among the 29 codons, most had an A/U at the third nucleotide position, which is considered to be due to the high A/T content found in most plastid genomes [53,59]. Unlike other species, *C. coreana* had an RSCU value of more than one for UCC and less than one for UCA among the codons encoding the amino acid serine (Table S3). This phenomenon of changing codon bias can be explained by complex factors such as genes and mutations selected during long-term adaptation to the environment and evolution [60]. In particular, *C. coreana* is an endemic species distributed only in the Republic of Korea, and it is considered to be a phenomenon that occurred after being isolated in the Republic of Korea for a long time and undergoing adaptation and evolution.

### 4.2. Divergence Hotspots of Corylopsis

SSRs are useful molecular markers for distinguishing species and are used to identify phylogenetic relationships within taxa because of their high degree of polymorphism [61,62]. A total of 73 (*C. multiflora* var. *nivea*) to 106 (*C. spicata*) SSRs were found in the genus *Corylopsis* (Figure 5a). Mononucleotide SSRs (78.33%) were the most common, followed by dinucleotide SSRs (10.05%), tetranucleotide SSRs (6.09%), trinucleotide SSRs (3.27%), and pentanucleotide SSRs (2.26%) (Figure 5a). SSRs consisting of A and T were the richest: the ratio of A/T nucleotide was overwhelmingly high in mononucleotide SSRs (72.60–80.19%), AT/AT in dinucleotide SSRs (7.55–10.59%), and AAAT/ATTT in tetranucleotide SSRs (1.89–4.11%) (Table S4). In the SSRs of many plants, poly A and poly T occur relatively more frequently than poly G or poly C, which is consistent with the SSR repetition results of *Corylopsis* identified in this study [45,63,64,65].

Repetitive elements like palindromic, forward, and reverse repeats, as well as complementary sequences, exert significant influence on genetic organization. They serve as valuable molecular markers for identifying phylogenetic relationships or distinguishing between species [46,66]. In this study, the repeat sequences of 10 plastid genomes, including those of *Corylopsis,* were searched (Figure 5b). In all species, forward and palindromic repeats accounted for more than 90% of the repeats. One or two reverse repeats were found in the remaining species, with the exception of one (*C. multiflora* var. *nivea*), and complementary repeats were found in only three species (*C. spicata*, *C. coreana*, and *C. microcarpa*). The length of most repeats was more than 30 bp and less than 50 bp, which is similar to the repeat results of Hamamelidaceae species found in a previous study (Table S5 and [3]).

A phylogenetically useful region can be selected through nucleotide diversity analysis, which can provide information on divergent hotspots in plastid genomes [20,67]. The nucleotide diversity (Pi) of the CDS, tRNA, rRNA, introns, and intergenic spacers was calculated (Figure 6). The Pi value of the IR regions was lower compared to that of the SC regions, suggesting a higher level of conservation in the IR regions than in the SC regions [64,67,68]. Most of the regions with high Pi values were non-coding regions such as intergenic spacers or introns, and the region with the highest Pi value was *rpl33*_*rps18* (Figure 6). This suggests that coding regions exhibit greater conservation compared to non-coding regions. In the coding region, *ndhE* exhibited the highest Pi value (Table S6). These selected regions can be useful molecular markers for phylogeny at the genus level, such as in DNA barcoding.

### 4.3. Phylogenetic Relationships within Corylopsis

The plastid genome has features such as a small and simple structure, well-conserved gene content and arrangement compared to the mitochondrial and nuclear genomes, and uniparental inheritance, which are considered informative and valuable for understanding evolutionary biology [69,70]. Several studies have conducted phylogenetic analyses of Hamamelidaceae using genes. Li et al. [9] identified the phylogeny of the *Corylopsis* complex (*Corylopsis*, *Distylium*, *Eustigma*, *Fortunearia*, and *Sinowilsonia*) using morphological features and internal transcribed spacers (ITS). Shi et al. [71] suggested the phylogeny of Hamamelidaceae based on ITS regions and 5.8 S coding regions of the nuclear genome and confirmed that the genus *Corylopsis* forms a monophyletic group with the genus *Loropetalum*. Wang et al. [3] used plastid genomes to confirm the phylogenetic relationships of genera belonging to Hamamelidaceae. However, studies conducted to date have examined the phylogenetic relationships of *Corylopsis* and other genera, and no study has been conducted to identify the phylogenetic relationships within *Corylopsis* using plastid genomes. This study outlines the proposed phylogenetic relationships of *Corylopsis*, utilizing concatenated protein-coding genes.

Three new plastid genomes of *Corylopsis* species were produced based on NGS using Illumina sequencing, and the systematic relationships of *Corylopsis* were identified based on the protein-coding genes of the plastid. Similar to previous studies, Hamamelidoideae, including *Corylopsis*, were confirmed to be monophyletic, and the genus *Loropetalum* is sister to *Corylopsis* [3,72,73]. *Loropetalum* is characterized by its colorful and red flowers, leathery elliptical leaves, and often evergreen habit [74]. In contrast, *Corylopsis* is known for its pendulous catkin-like clusters of small, yellowish flowers, serrated deciduous leaves, and a more upright growth habit [1]. Within the genus *Corylopsis*, the earliest branched species was *C. multiflora* var. *nivea,* which is a variety of *C. multiflora* characterized by glabrous young branches, leaves, peduncles, and short stamens and is endemic to Mt. Fuji, China (Figure 7) [2,24]. The branched species are *C. microcarpa,* distributed in China, and *C. coreana*, which is endemic to the Republic of Korea [21]. Previously, *C. coreana* was considered as *C. gotoana* var. *coreana*; however, they are distinguished by the presence or absence of hair on the lower surface of leaves and the number of flowers per inflorescence. Because a prominent difference between the two species was observed in the results of the phylogenetic tree, considering them as independent species is reasonable [21,75]. *C. pauciflora*, *C. spicata*, and *C. gotoana* form a clade, and all share the common feature of being distributed in Japan. *C. pauciflora* is distinguished by its notably smaller leaves (less than 6 cm) compared to other species (approximately 10 cm), and its inflorescence is also characterized by its short size, consisting only of one to five flowers [1]. Additionally, *C. spicata* is morphologically distinguished by its filaments being bright red, whereas those of other taxa in the genus are typically yellow or white [1]. Four species of *Corylopsis* are known to be distributed in Japan, three of which were included in this study. The adjacent relationships between the three species and the formation of a monophyletic group suggest that speciation occurred in Japan. Yamanaka et al. [7] also suggested the possibility that independent speciation may have occurred in areas where *Corylopsis* species in Japan became refugia under the influence of Quaternary climate change [7]. *C. velutina*, *C. glandulifera*, and *C. sinensis* formed a clade, all of which were distributed in China. The plastid genome of *C. sinensis* obtained in the present study formed a clade with that previously listed in GenBank (MZ590567). Most of the distribution area of *Corylopsis* is occupied by China, but five species distributed in China were included in this study. A phylogenetic study involving the species distributed in China is essential for understanding the biogeographical evolution of *Corylopsis*. The correlation between the geographical distribution and clade formation of phylogenetic trees can usually be estimated using fossil data such as divergence time and migration route. Kim et al. [22,23] identified the biogeographical history of the Northern Hemisphere by inferring the migration routes and divergence times of Melanthiaceae species based on fossil data. Although this study did not investigate the biogeographical history using fossil data, it is considered that their evolutionary history and distribution may be related, as clades within *Corylopsis* are formed by their distribution.

In this study, 9 of 25 species belonging to the genus *Corylopsis* were analyzed to confirm the lineage within *Corylopsis* and provide information on the new plastid genome. This is significant in that it is the first study to compare the plastid genomes of species belonging to the genus *Corylopsis* and to explore the phylogenetic relationship between taxa. However, there is a limitation in that there is a node with a low support value, and the phylogenetic relationship of the entire genus *Corylopsis* cannot be confirmed. If more species are added and analyzed, the limitations of this study will be resolved, and the various pieces of information presented in our results will serve as the foundation for identifying the phylogenetic history of Hamamelidaceae in the future.

## 5. Conclusions

For the first time, our study performed a comprehensive comparative analysis based on the plastid genome of *Corylopsis* and phylogenetic relationships. We also provided information on the plastid genomes of the three species within *Corylopsis*. The *Corylopsis* plastome has the quadripartite structure of a typical angiosperm plastid genome, ranging from 158,996 bp to 159,507 bp. It comprises 79 protein-coding genes, 30 tRNAs, and 4 rRNA genes. Through various plastome structure analyses, the plastid genome structure of *Corylopsis* was similar and well-conserved. Repeat and nucleotide diversity analyses were performed to search for divergent hotspots that could be used as molecular markers. The number of SSRs in *Corylopsis* ranged from 73 to 106, and mononucleotide SSRs accounted for the largest proportion. More than 90% of the repeats were composed of forward and palindromic repeats, and most repeats were 30–50 bp in length. A phylogenetically useful region was identified using nucleotide diversity analysis. The Pi values of the SC regions were higher than those of the IR regions, and the highest Pi value was *rpl33*_*rps18* intergenic spacer region. Phylogenetic analysis using the protein-coding genes of the plastid genome confirmed that the genus *Corylopsis* is a monophyletic group and that its sister is a genus of *Loropetalum*. In this study, the phylogenetic relationships within *Corylopsis* were demonstrated, which will be helpful for identifying the phylogeny of Hamamelidaceae in the future.

## Figures and Tables

**Table 1:** Comparison of the plastome features of *Corylopsis* and related taxa.

Taxa	Length (bp) and GC Content (%)				GenBank Accession Number
	LSC	SSC	IR	Total	
*Corylopsis sinensis* *	88,149 (36.1%)	18,704 (32.7%)	26,283 (43.1%)	159,419 (38.0%)	PP273282
*Corylopsis sinensis*	88,152 (36.1%)	18,701 (32.7%)	26,283 (43.1%)	159,419 (38.0%)	MZ590567
*Corylopsis glandulifera*	88,134 (36.1%)	18,702 (32.6%)	26,283 (43.1%)	159,402 (38.0%)	MZ642354
*Corylopsis velutina*	88,146 (36.1%)	18,702 (32.7%)	26,283 (43.1%)	159,414 (38.0%)	MZ823391
*Corylopsis gotoana* *	88,164 (36.1%)	18,702 (32.7%)	26,284 (43.1%)	159,434 (38.0%)	PP273280
*Corylopsis spicata*	88,243 (36.1%)	18,716 (32.7%)	26,274 (43.1%)	159,507 (38.0%)	MK942341
*Corylopsis pauciflora* *	88,097 (36.1%)	18,700 (32.7%)	26,283 (43.1%)	159,363 (38.0%)	PP273281
*Corylopsis coreana*	88,166 (36.1%)	18,692 (32.7%)	26,270 (43.1%)	159,398 (38.0%)	MG835449
*Corylopsis microcarpa*	88,185 (36.1%)	18,693 (32.6%)	26,280 (43.1%)	159,438 (38.0%)	MZ642356
*Corylopsis multiflora* var. *nivea*	87,895 (36.1%)	18,672 (32.6%)	26,213 (43.1%)	158,993 (38.0%)	MW043717
*Loropetalum chinense*	88,160 (36.1%)	18,770 (32.7%)	26,257 (43.1%)	159,444 (38.0%)	NC_060831
*Loropetalum subcordatum*	88,216 (36.1%)	18,494 (32.7%)	25,998 (43.1%)	158,706 (38.0%)	NC_037694
*Distylium myricoides*	87,847 (36.2%)	18,780 (32.5%)	26,233 (43.1%)	159,093 (38.0%)	NC_059883
*Distylium racemosum*	87,863 (36.2%)	18,782 (32.5%)	26,231 (43.1%)	159,107 (38.0%)	NC_059886
*Hamamelis mollis*	88,301 (36.1%)	18,762 (32.5%)	26,334 (43.1%)	159,731 (38.0%)	NC_037881
*Sinowilsonia henryi*	87,507 (36.4%)	18,768 (32.8%)	26,233 (43.1%)	158,741 (38.2%)	NC_036069
*Mytilaria laosensis*	89,016 (35.9%)	18,127 (32.8%)	26,399 (43.1%)	159,941 (37.9%)	NC_048997
*Rhodoleia championii*	88,144 (35.8%)	18,131 (32.3%)	26,420 (42.9%)	159,115 (37.7%)	NC_045276
*Liquidambar styraciflua*	88,891 (36.1%)	18,977 (32.4%)	26,441 (43.0%)	160,750 (37.9%)	NC_046938
*Liquidambar orientalis*	88,882 (36.1%)	18,947 (32.4%)	26,471 (43.1%)	160,771 (37.9%)	NC_046937

* Sequenced in this study.

## Data Availability

The associated BioProject number is PRJNA1066397. SRA numbers are SRR27630243 (*Corylopsis sinensis*), SRR27630244 (*C. pauciflora*), and SRR27630245 (*C. gotoana*). The biosample numbers are SAMN39487248 (*C. gotoana*), SAMN39487249 (*C. pauciflora*), and SAMN39487250 (*C. sinensis*). The new plastome sequences are available in GenBank: PP273280 (*C. gotoana*), PP273281 (*C. pauciflora*), and PP273282 (*C. sinensis*).

## Supplementary Materials

The following supporting information can be downloaded at: https://www.mdpi.com/article/10.3390/genes15030380/s1, Figure S1: The complete plastid genomes assembled in this study; Table S1: List of samples for total DNA extraction; Table S2: List of annotated genes in the plastid genome of *Corylopsis*; Table S3: Relative synonymous codon usage (RSCU) analysis of protein-coding genes in 10 *Corylopsis*; Table S4: The number of simple sequence repeats (SSRs) identified within the plastome of *Corylopsis*; Table S5: List of Repeat analysis of *Corylopsis*; Table S6: Nucleotide diversity (Pi) of 10 *Corylopsis*.
